# Immunohistochemical characterization of pancreatic duodenal homeobox protein-1, neurogenin-3, and insulin protein expressions in islet-mesenchymal cell *in vitro*: a morphochronological evaluation

**DOI:** 10.22038/IJBMS.2018.26688.6536

**Published:** 2018-11

**Authors:** Juziel K Manda, Venant Tchokonte-Nana

**Affiliations:** 1 Islet and MSK Research Group, Anatomy and Histology, Department of Biomedical Sciences, Faculty of Medicine and Health Sciences, Stellenbosch University, Tygerberg, Western Cape, South Africa

**Keywords:** Co-culture, Duct ligated pancreas, Insulin, Islet, MSC, NeuroG3, Pdx1, Transplantation

## Introduction

There is a pressing need to identify new sources of donor islets that are functionally capable of reverting hyperglycaemia in diabetes mellitus. A rodent model of surgical insult on adult pancreatic duct creates degeneration of the exocrine acinar cells while the endocrine pancreas remains unaffected in the portion distal to the ligation point (1, 2). The destruction of exocrine tissue is followed by the proliferation of fibroblastic mesenchymal cells (MSCs) around the degenerating acini, leading to the proliferation of ductal structures (3). Small clusters of Neurogenin-3 (Ngn3) positive islet cells accumulate close to the metaplastic ducts and later migrate to become interspaced within the enlarged fibrous mesenchymal septa and adipose tissue which results in a larger association of beta cells in the tail portion of the pancreas (2). This observation has compelled many authors (2, 4–6) with additional compensatory growth in response to increased demand to suggest that injury to the pancreas by means of surgical ligation activates a neogeneic program in small ductules similar to the foetal pancreas, which could be a potential source of donor islet for transplantation. Later studies (7–9) observed considerable amounts of Ngn3 positive islet cells within mature islet cells in injured mice pancreata. This could imply that a possible pool of cells exist outside the ducts where neogenesis may occur (9). 

Although ample evidence (10, 11) suggest that beta cell regeneration is induced by tissue injury, the extent of cell-cell interactions and the molecular mechanism by which this process is regulated are currently unclear. Gittes *et al.* (12) reported the importance of epithelial-MSC interactions on lineage-specific morphogenesis in the normal pancreas. Similarly, signals from MSCs have been shown to control proliferation of pancreatic precursors in a mouse embryo (13). In chicks, mesenchymal tissue is significant for directing a pancreas-specific fate (14); while in foetal rats, intestinal MSCs combined with endodermal cells induce the differentiation of the main epithelial cell types including endocrine cells (15), suggesting the key role that MSCs play in the differentiation of endocrine cells (16, 17). Signals from MSCs may therefore direct islet cells toward a beta-cell neogenesis or may maintain the requirement or the direction of proper timing of differentiation, proliferation, or migration of beta cells. Despite its role during pancreatic development, there is no convincing evidence for the presence of beta cell neogenesis in the adult normal pancreas (18)**.** In adult mice, MSCs include endothelial cells, pancreatic fibroblasts and in smaller numbers, stellate and vascular smooth muscle cells (19). Culture conditions for long-term self-renewal of human multipotent pancreatic progenitors similar to specialized adult pancreatic cells have been developed (20)**.** Co-culture of islets of Langerhans with MSCs from various sources has a potential to revert hyperglycaemia following transplantation, but the resulting insulin-producing cells (IPCs) express low levels of endocrine progenitor genes necessary to promote islet function and sustain isograft survival. In injured adult pancreas models, it is currently not known whether MSCs alone regulate the neogeneic program to increase beta-cell mass. Tchokonte-Nana (3) reported the presence of fibroblast mitotic cells in pancreatic tissue 6 hours after surgical duct ligation had taken place indicating that MSCs replication precedes endocrine remodelling (21). Thus, this study aimed to investigate the morphochronology of a co-culture of Islet with MSCs isolated from the splenic portion of injured adult pancreata and characterize pancreatic duodenal homeobox protein-1 (Pdx1), Ngn3 and insulin protein expressions to establish the fate of their interactions. Insulin production and secretion as a result of Islet-MSC interactions could play an important role in the cell replacement therapy of DM.

## Materials and Methods

Healthy adult male Wistar rats (250-300 g) were obtained from the Animal Unit of the Faculty of Medicine and Health Sciences, Stellenbosch University (Cape Town, South Africa). The use of animals and the study protocol were approved by Stellenbosch University Animal Care and Use Committee (SU ACUC): ethics number (SU-ACUM13-00036). 

***Surgical procedure and tissue preparation***

Animals (n=16) were anaesthetized by inhalation of Isofor (Safeline Pharmaceuticals (Pty) Ltd, Roodepoot, South Africa) and a midline laparotomy was performed as described by Tchokonte-Nana (3). The post splenic duct of the pancreas was ligated in experimental animals (PPDL) (n=8), and the control animals were sham operated (SOC) (n=8) while the pancreata were gently touched with a cotton swab for 30 seconds without ligation. The splenic part of the pancreas in the PPDL and SOC was excised 24 hr following surgery and placed on Hanks’ balanced salt solution (HBSS) to clean out blood tissue, in preparation for cell isolation procedures. 

***Cell isolation and separation***

The excised pancreas was injected with 2 ml (1.5 mg/ml) Collagenase A (Sigma) and sliced into 2 mm pieces 5 min later and placed in conical tubes containing 10 ml (1.5 mg/ml) Collagenase A. The pancreas tissue was allowed to digest at 37 ^°^C in the collagenase solution for 30 min followed by Ficoll (Sigma) density gradient separation.

***The islets***

Islets were hand-picked under a Nikon microscope (Nippon Kogaku KK, Japan). The isolation of islets was based on the assessment of their purity using Dithizone (Sigma-Adrich) and their quality were confirmed by visual assessment of their morphology (16, 22). Islets were then distributed into groups of 50, washed in HBSS twice before re-suspension in RPMI1640 (GIBCO) at 37 ^°^C in a humidified atmosphere of 95% air and 5% CO_2 _ready for culture.

***Mesenchymal stromal cells***

MSCs were isolated from the remaining islet-depleted collagenase digest cultured in T25 flasks. Isolation was based on their ability to adhere to plastic dishes and differentiate to adipocytes and osteocytes as described previously (23). Supernatant was removed 48 hr later and adherent MSCs were maintained as a monolayer at 37 ^°^C in humidified air (95%) and 5% CO_2_ in Dubelco’s modified Eagle medium (DMEM) (GIBCO) supplemented by 1% (vol/vol) penicillin-streptomycin (GIBCO) and 10% foetal bovine serum (FBS). The emerging fibroblastoid-like cells (MSCs) attached to the surface were passaged every third to fourth day until 80-90% expansion before further co-culturing with isolated islets. These MSCs were exposed to the co-culture with no further characterization for the precise stem-cell identity.

***Co-culture of islets and MSCs***

Islets were cultured with or without MSCs isolated from PPDL and SOC tissues. As previously described (16), approximately 150,000 MSCs of passage 3 were seeded on each of the Cell Star 15 mm Petri dishes (Greiner Bio-One). Fifty fresh islets were seeded directly on the MSC monolayer and cultured for 24 hr to form a confluent monolayer for the direct contact islet-MSC monolayer co-culture system. When sub-culturing, adherent composite islet-MSC cells on Petri dishes were mildly trypsinized with a pre-warmed 0.05% trypsin-EDTA to detach the cell aggregates (islet/MSC). All islet aggregates were pipetted, counted and transferred into a new T25 culture flask. The remaining MSCs in suspension were thereafter, sub-cultured in a 1:5 split ratio by taking 1 ml of MSC suspension and adding it to a new T25 culture flask containing 4 ml of complete fresh medium to make a total of 5 ml of cell suspension. Twenty-five islets were then seeded on T25 culture flasks containing 5 ml of MSCs suspension in RPMI-1640 medium supplemented by 1% (vol/vol) penicillin-strepotomycin (GIBCO) and incubated at 37 ^°^C in a humidified atmosphere of 95% air and 5% CO_2_. The culture medium was changed every 2 days. The cultured cells were stained with dithizone and were morphologically assessed on designated times. 

***Immunnocytochemistry***

For immunostaining, the cultured cells with or without MSCs were washed with PBS (3x5 min) and cytospun onto positively charged slides (BIO-SCAN). Tissue slides were fixed in 4% PFA for 30 min at room temperature and processed for immunostainings. Prior to staining, tissue slides were exposed to antigen retrieval procedure and were later immersed in an antigen retrieval buffer (100 mM Tris, 5% (w/v) urea, pH 9.5) (Leica) and placed in a water bath at 95 ^°^C for 10 min. Tissue slides were then blocked with 2% BSA for 1 hr at room temperature. Double immunolabeling was applied to the slides using the following combination of antibodies: polyclonal rabbit anti-Pdx1 and the polyclonal rabbit anti-Ngn3; anti-Pdx1 and the monoclonal mouse anti-insulin; and anti-Ngn3 and anti-insulin. These antibodies (ABCAM) were used as primary antibodies and were diluted as per manufacturer’s instructions. The secondary antibodies used were the Alexa Fluor 594- conjugated goat anti rabbit (ABCAM) and the Alexa Fluor 488-conjugated goat anti rabbit (ABCAM) diluted at 1: 800 each. 

The nuclei were counter-stained with 4’-6- diamidino-2-phenyl-indole (DAPI, Sigma) at 300 µg/ml. Slides were observed and images captured under a Nikon Eclipse Fluorescent Microscope (TE2000-S, Nipon Kogaku K.K., Japan) for further analysis.

***Morphometry analysis***

Conjointly with the assessment of islets in culture at designated times, digital images of immunostained slides were segmented and features of positive-labelled cells for specific antigens were programmed using the auto measurement software of NIS-Elements (Basic Research [BR] 3.2). About 50 islets were analysed per culture condition. The cultured cells were quantified as follows: 

• The surface area of islets in islet culture with or without MSCs were measured at day 0 (start of culture), day 15 and 25 and expressed in µm^2^.

• The fraction of insulin^+^/Pdx1^+^ cells in islet culture with or without MSCs was calculated at day 1, 3, 5 and 7 and expressed as %.

The proliferation index (PI), which is essential in quantifying islets development in culture with or without MSCs, was calculated as a value without unit at day 3 to 12 using the following formula:

$\mathrm{PI}=\frac{\mathrm{surface area of Pdx1}+\mathrm{cells}}{\mathrm{number of Ngn3}+\mathrm{cells count}}$

Also, the efficacy index of Ngn3 expression (PhI), which is essential in determining Ngn3^+^ cell-dependent maturity and function of beta cells in islet culture with or without MSCs, was calculated as a value without unit at day 24 to 28 using the following formula:

$\mathrm{PhI}=\frac{\mathrm{fraction of insulin}+\mathrm{cells}}{\mathrm{surface area of Ngn3}+\mathrm{cells}}$

**Figure 1:**
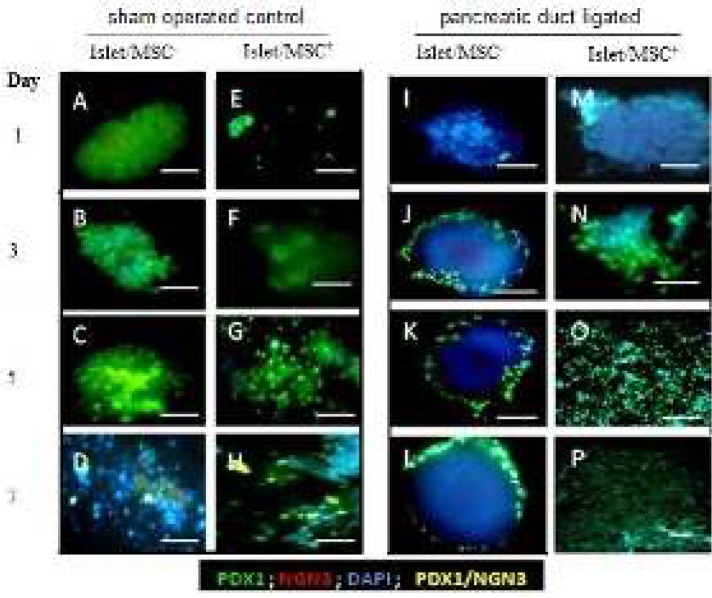
Expression of pancreatic duodenal homeobox protein-1 and neuroginin-3 during early development of islets cultured by direct contact with or without mesenchymes. Immunocytochemistry analysis of whole mount islets cells isolated from PPDL and SOC and cultured without (A-D) for PDL and I-L for SOC, or with (E-H) MSCs for PDL and M-P for SOC. Islet cells development was evaluated by anti-Pdx1 (green) and anti-Ngn3 (red). PDX1/NGN3 (orange). Nuclei were counter-stained in blue with DAPI. ), Scale bar = 100 µm

**Figure 2:**
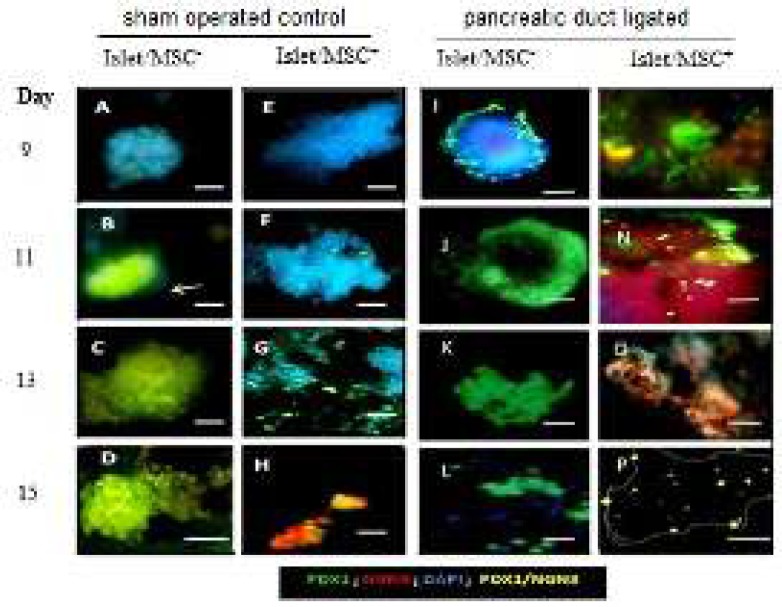
Expression of pancreatic duodenal homeobox protein-1 and neurogenin-3 during the second week of development of islets cultured by direct contact with or without mesenchymes. Islets and mesenchymal cells (MSCs) were isolated from SOC and PPDL tissues respectively and cultured with (islets+ MSC-, islets/ MSC+) for different periods. Immunocytochemistry analysis of PPDL islets cells cultured without (A-D) or without (A-D) with (E-H) for 9 (A and E), 11 (B and F), 13 (C and G) and 15 (D and H) days of culture. I-P: Immunocytochemistry analysis of 24 hr SOC islets cells cultured without (I-L) or with (M-P) MSCs for same period of time. Islet cells development was evaluated by anti-Pdx1 (green) and anti-Ngn3 (orange/red). Nuclei were counter-stained in blue with DAPI. Bar = 100 µm

**Figure 3:**
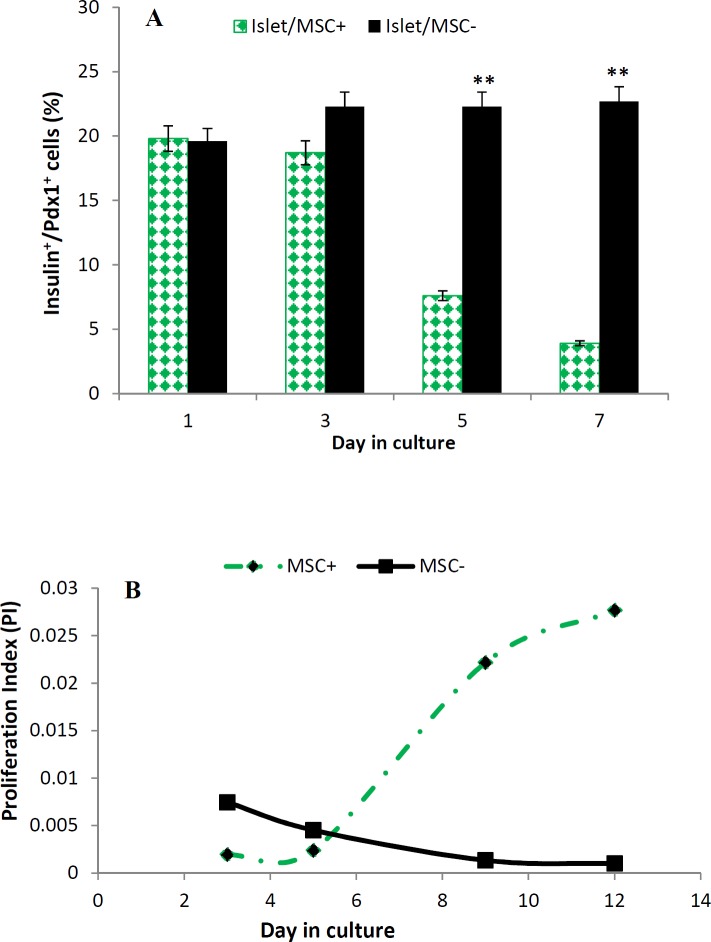
A) A bar-graph representing insulin+/pancreatic duodenal homeobox protein-1+ co-expression during the first week of development of islet/mesenchymal cell cultures of duct ligated pancreata. Quantification of absolute surface areas occupied by Insulin+Pdx1+cells that developed over 3, 5, 7 days of culture with or without mesenchyme. Forty (40) to 50 islets were analysed for each condition. Data are means±SE. **P<0.05 (P=0.0481), Scale bar =100 µm. B) A trend graph showing the proliferation index of cells in islet/mesenchymal cell cultures of duct ligated pancreata during 2 weeks of development. Forty (40) to 50 islets were analysed for each condition. Data are means ± SE. **P<0.05. Scale bar=100 µm

**Figure 4:**
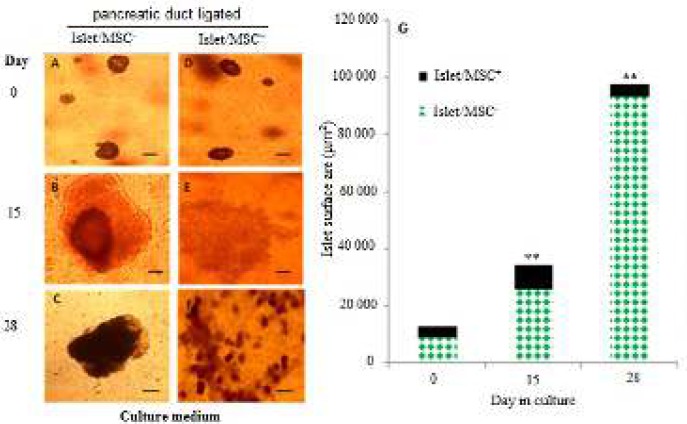
Representative growth in size of the islets in islet/mesenchymal cell cultures of duct ligated pancreata. Islets were grown for 28 days with or without MSCs. A and D: Islets at day 0 of culture with or without MSCs. B and E: Islets cultured for 15 days with or without MSCs. C and F: Islets cultured for 28 with or without MSCs. The surfaces of the islets after dithizone staining were marked and measured and areas of all islets were added up. G: Quantification of the total size of the islets before, after 15 and 28 days in culture in the absence or presence of MSCs. Thirty (30) to 40 islets were analysed in each condition. Data of mean values in µm^2^±SE are shown in the graph. **P<0.05

**Figure 5:**
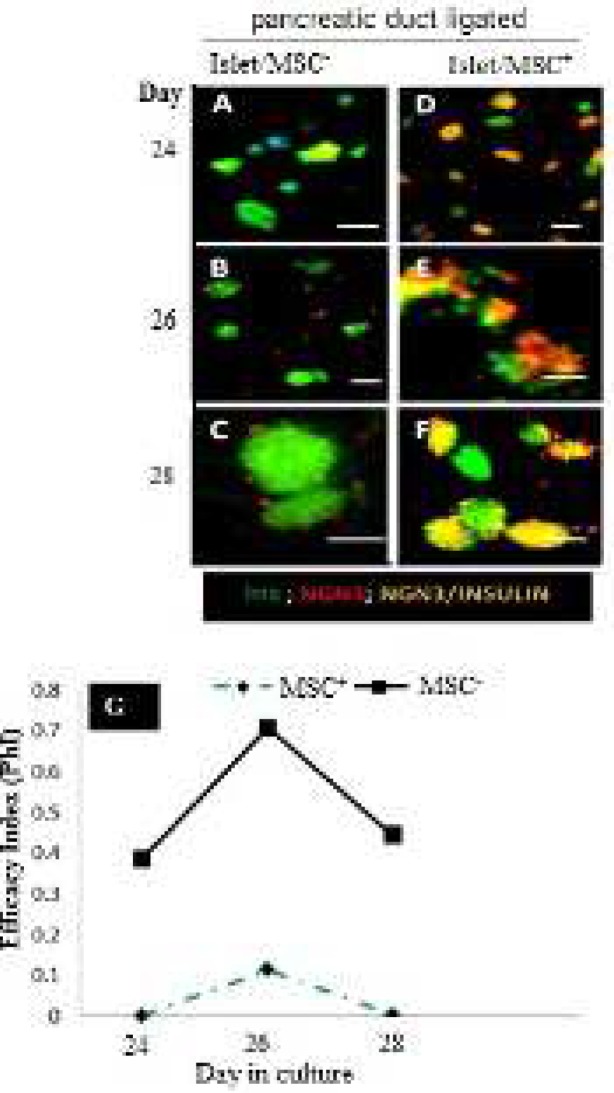
Expression of insulin and neurogenin-3 and the efficacy index of neurogenin-3 in islet/mesenchymal cell cultures of duct ligated pancreata. Immunocytochemistry analysis of islet cultures of PPDL tissues without (A-C) or with (D-F) MSCs. Islet cells development was evaluated by anti-Insulin (green) and anti-Ngn3 (orange/red). G: Quantification of the absolute surface areas occupied by insulin+cells and Ngn^3+ ^cells that developed over 24, 26 or 28 days of culture with or without MSCs. Forty (40) to fifty (50) islets were analysed for each condition. Data are means±SE. *P<0.05 (*P=*0.037). Scale bar=100 µm

All data were presented as Mean ± SEM. A statistical significance was determined using Student’s t-test for *P*-value ≤ 0.05.

## Results

This study presents the morphological changes of the islets cultured in direct apposition with or without MSCs from isolated cells of both the PPDL and SOC tissues over a period of four weeks. The protein expression of Pdx1, Ngn3 and insulin in islet/MSCs aggregates were assessed to establish the fate of their interaction. 

***First and second weeks of islets in culture with or without MSCs***

Islets cultured with or without MSCs (islet/MSc^-^ or islet/MSC^+^), using both the PPDL and SOC tissues, were positive for Pdx1 throughout the first seven days (Figure 1). As far as Ngn3^+^ cells, there were no detections throughout the first 7 days of islet/MSc^-^ and islet/MSC^+^ cultures in either SOC or PDDL. This observation possibly suggests that in the early hours of development of PPDL tissue, Ngn3 is induced by factors other than those arising directly from MSCs. 

Figure 2 shows further expression of Pdx1 until the end of the second week where Pdx1^+^ cells were seen in islet/MSC^-^ culture of both SOC and PPDL tissues. The expression of Pdx1 in these cells was down-regulated in the second week of islet/MSC^+^ culture of both PPDL and SOC tissues. In addition, Ngn3^+^ cells were observed in islet/MSC^+^ cultures of both SOC and PPDL tissues when compared to the islet/MSC^-^ cultures in the same period of the culture. 

The immunostaining analysis of the co-expression of Pdx1 and insulin in the first week is represented in Figure 3A. Insulin positive cells in islet/MSC culture, often co-expressing with Pdx1, continued to be expressed in significantly high levels compared to islet/MSC^+^ culture (*P*<0.05) suggesting that when islets are cultured without MSC^-^, proliferating beta cells still maintain their mature phenotypes even after two weeks of culture. However, in islet/MSC^+^ culture, co-expression of insulin positive cells with Pdx1 gradually decreased. This result may suggest that these proliferating cells began to adopt a less differentiated or different phenotype in the presence of MSCs.

A double immunofluorescence analysis confirmed co-localizations of Pdx1 and Ngn3 in islet/MSC^+^ of SOC tissues on day 7, and this co-expression was further observed at days 13 and 15 in the second week of culture. While in islet/MSC^-^ and islet/MSC^+ ^cultures of PPDL tissues, Pdx1^+^/Ngn3^+^ cells were observed from days 11 to 15 (Figure 2). The proliferation index (PI) in PPDL tissues (Figure 3B), shows a decreasing trend of the proliferation in islet/MSC^-^ culture, whereas islet/MSC^+^ culture had a significant increase in the trend of the PI from day 5 up to day 12. Then, this evidence supports the implication of MSCs in the islet cell proliferation. 

***Third and fourth weeks of islets in culture with or without MSCs***

Further culturing was carried out in PPDL tissues for up to the fourth week with or without MSCs to assess the growth and maturation of islets, and evaluate Ngn3 efficacies in beta cell function of islet/MSC cultures. At the start of the third week of the islet cell culture with or without MSCs, differences in rates of proliferation were observed (Figure 4 B, E). In the islet/MSC^-^ culture, the size of islet did not increase any further between day 15 and 28 (Figure 4 A-C). Even after passaging every 6 days, islets failed to reach confluence. Even so, a few new small islet-like cell aggregates in islet/MSC^+^ culture began to form within expanding islet cells from day 17 and this growth process continued up to day 28 resulting into

numerous but small islet-like clusters (Figure 4 D-F). Quantitatively, in the presence of MSCs (islet/MSC^+^), islet sizes continued to increase such that by day 28 there was a significant difference in the size of islets compared to islet/MSC^-^ cultures (Figure 4 G, *P *= 0.001). In short, our data strongly suggest that the presence of MSCs promotes expansion of islet cells resulting in a formation of more islet-like aggregates. 

Further quantitative immunofluorescence analysis shows that on day 24, significantly higher levels of Ngn3^+^ cells were detected in islet/MSC^+^ when compared with islet/MSC^-^ culture (Fig. 5 [A compared with D], *P *= 0.037). As a result, continued expression of high levels of Ngn3^+^ cells was detected up to day 28 of culture in islet/MSC^+^ (Figure 5 E-F)**,** possibly suggesting that high numbers of Pdx1^+^ cells have differentiated into Ngn^3+^ cells. To put it another way, these results therefore suggest that, in addition to promoting the expansion of Pdx^1+^ islet cells, MSCs also maintain Ngn3 expression in the proliferating islet cells. In spite of this, insulin expression was significantly higher in islet/MSC^-^ compared to islet/MSC^+^ (Figure 5 [A-C compare with D-F], H). More so, insulin expression gradually increased in islet/MSC^+^ cultures towards the end of the fourth week, but was interestingly low compared to islet/MSC^-^ culture (Figure 5 C-F). 

The analysis of the co-expression of Ngn3 and insulin in PPDL tissues revealed a higher percentage of cells located at the centre of islets co-expressing insulin and Ngn3 up to day 28 in in islet/MSC^+^ culture (Figure 5 D-F). In islet/MSC^-^ culture, single nuclear Ngn3^+^ cells were detected in the periphery of the islets from day 26 to 28 (Figure 5 B-C), mainly suggesting that Ngn3 was possibly expressed by non-insulin-producing cells. Strongly enough, insulin^+^/Ngn^3+^ co-expressing cells observed on day 28 in islet/MSC^+^ culture support the role of MSCs in beta cell maturation and function. Furthermore, the efficacy index (PhI) (Figure 4 G) shows that irrespective of the levels of the expression of insulin in islet cells in islet/MSC^-^ or islet/MSC^+^ cultures, the PhI of Ngn3 dropped from day 26 in both islet/MSC^-^ and islet/MSC^+^ cultures, presenting the necessity of Ngn3 down-regulation in insulin production. Indeed, we postulate that islet/MSC^-^ (islets cultured alone) may yield mature insulin producing cells from non-beta-cells.

## Discussion

This study attempted to develop an *in vitro* model in which islet cells isolated from a splenic portion of a duct ligated rat pancreas (PPDL) were allowed to develop with or without mesenchymal cells (MSCs) for four weeks. The fate of such islet/MSC *in vitro* interaction was established by chronologically evaluating an immunohistochemical characterization of the protein expression of Pdx1, Ngn3 and insulin. The observation in the first two weeks of culture suggests that in the culture without MSC^-^, proliferating beta cells maintained their mature phenotype by co-expressing insulin and Pdx1 even after two weeks of culture. In contrast, in islet/MSC^+^ culture, the co-expression of insulin with Pdx1 was found to gradually decrease, suggesting that these proliferating cells began to adopt a less differentiated or different phenotype in the presence of MSCs. The presence of Ngn^3+^ cells at the end of the second week in both SOC/MSC^+^ and PPDL/MSC^+^ clearly indicates the direct implication of MSCs in the early hours of development (Figure 2). Apart from this, a significant increase in the trend of the proliferation index (PI) from day 5 up to day 12 of islet/MSC^+ ^culture was proven evidence, implicating MSCs in the islet formation (3, 12)and the way in which endocrine, acinar and ductal cell lineages are generated from the embryonic foregut. Notably, these results support previous authors (7–9) who suggested the existence of a site of progenitor cells within mature islets in injured mice pancreata, contradicting some viewpoints in the literature (18). 

From the immunostaining analysis in both cultures with or without MSCs, activation of Pdx1 in islet cells was observed. Moreover, by the end of the second week, Pdx1^+^ cells in the islet/MSC^+^ culture expanded up to 80% confluence more than in the islet/MSC^- ^culture. Owing to this, Pdx1 is regarded as a key transcription factor involved in early pancreatic development, differentiation of insulin-producing beta cells and maintenance of mature beta cells (24). In mature beta cells, Pdx1 is one of the key genes that regulate the transcription of insulin (24), GLUT2 (25), Glucokinase (26) and Nkx6.1 (27) genes. In the presence of MSCs, Pdx1 was highly activated during the first week of culture in this study; and whether Pdx1 regulated proliferation of beta cells or maintained the identity of mature beta cells in cultures was not clearly understood. Actually, Pdx^1+^ cells expanded relatively higher in all cultures during the first week (Figure 1) and this was followed by high levels of Ngn^3+^ cells in islet/MSC^+^ culture at the end of the second week of development (Figure 2). When islets were cultured alone, Pdx1 was also highly activated and often co-expressing with insulin during the first week, while Insulin/Pdx1 co-expression decreased gradually in islet/MSC^+^ culture when compared to islet/MSC^-^ culture (Figure 3A). In other words, when islets are cultured alone, proliferating islet cells maintain their differentiated phenotypes by continuously co-expressing Pdx1 and insulin; while in cultures with MSCs the embryonic pathway of differentiation in cultured islet cells was activated. 

The mechanism by which MSCs reactivate the neogeneic pathways and regulate the proliferation and re-differentiation of these mature endocrine cells is still not clearly understood. The continuous increase in the size of islets in islet/MSC^+^ culture was significantly different to islet/MSC^-^ cultures (Figure 4 G, *P *= 0.001), where the islet growth did not improve from the second to the fourth week of development. This outcome shows that MSCs do not only promote expansion of islet cells but also maintain Ngn3 expression in the proliferating islet cells. Particularly, the presence of insulin^+^/Ngn^3+^ cells observed on day 28 in islet/MSC^+^ culture confirms the role of MSCs in beta cell maturation and function, suggesting an alternative source of adult beta cells (20) as donors for the cell replacement therapy in diabetes. Further investigation showed a drop of the efficacy of the Ngn3 in both islet/MSC^- ^and islet/MSC^+^ cultures by the end of the fourth week, signifying the necessity of Ngn3 down regulation in insulin production (28). Thus, the presence of high levels of insulin^+^ cells at the centre of the islets coupled with the peripheral Ngn^3+^ cells in islet/MSC^-^ culture strongly suggests a production of mature insulin by non-beta-cells within the islet, providing supports to a previous research (29). These results therefore suggest that activation of insulin production from non-beta cells may not need direct signals from MSCs but that a surgical ligation may activate a neogeneic program in pre-existing beta cells in the mature pancreas. Indeed, maintenance of Ngn^3+^ cells by MSCs may prevent premature differentiation of endocrine cells and allow islet cells ample time for expansion as well as to increase the number of re-differentiated cells into mature insulin producing beta cells. These are important findings of this study. Unfortunately, the origin of the Ngn^3+^ cells could not be explained by this study as only snapshot immunocytochemical analyses were used. Also, lineage tracing studies are required to investigate whether MSCs actually stimulate Ngn3 expression in mature de-differentiated islet cells or stem cells located within the islets. 

## Conclusion

In brief, based on the current findings and that of other published pancreas data, this study has demonstrated that *in vitro*, activation of insulin production from non-beta cells in the islet may not be induced by direct signals from MSCs. Undoubtedly, the down-regulation of Pdx1 and the maintenance of Ngn^3+^ cells in islet/MSCs cultures justify the implication of mesenchymal tissues surrounding pre-existing islets and proliferating ductules in islet regeneration and function. Therefore, the use of extracellular factors – factors from MSCs that stimulate tissue-intrinsic progenitor cell phenotypes could be an important step towards generating functional beta cells for transplantation as a therapeutic measure for diabetes mellitus. 

## Study Limitations

In this study, MSCs were characterized based on their morphology and there was no further characterization for the precise stem cell identity using some bimolecular assays such as the quantitative tests/analyses and the flow cytometry analysis.
